# Impact of the place on human health during pandemic and methods to enhance

**DOI:** 10.1007/s43995-022-00009-7

**Published:** 2022-11-22

**Authors:** Mohamed Abdelbary Wafa Mowafy

**Affiliations:** Department of Self-Development, Deanship of Preparatory Year and Supporting Studies, Imam Abdulrahman Bin Faisal University, P.O Box: 1982, Dammam, 31441 Saudi Arabia

**Keywords:** Earth energy, BioGeometry, Shape effect, Human body, Bioenergy

## Abstract

The spread of COVID-19 worldwide pushed many governments to impose curfews on citizens, that lead a major number of people to stay longer times at certain places in their homes. This is descriptive research, as this paper demonstrates the possibility of impact of the place on its occupant’s health, also it reviews the literature to present a framework to deal with that case in the future, whether there is a need for a new building or for just some modifications in existing ones. The research results showed that there is a certain impact of the place on the human physical and mental health, which results from one or both, the natural earth electromagnetic field and the shape design and proportions. In conclusion there are many methods and theories to deal with the energies of the place that have a negative impact on humans, one of them is to apply a regulatory energy that eliminates the negative impact of the existing energies, whether natural or artificial.

## Introduction

First appearance of COVID-19 was in November 2019 in China [1], it started to spread in China and then all over the world. World Health Organization (WHO) declared its spread as a pandemic on March 11th, 2020 [2]. Dr. Tedros Adhanom Ghebreyesus, the WHO Director-General, said it is not just a public health crisis, but it will touch every sector [3]. He also said that the number of cases outside China had increased 13-fold in two weeks, and he asked the governments to change the course of the pandemic by taking “urgent and aggressive action” [4]. That pushed many governments to impose curfews on citizens.

There is no doubt that the precautionary steps taken by the governments of many countries, by imposing curfews and keeping citizens at home for long hours, have affected the health of people, whether psychological, physical, or mental.

The word place in this research generally refers to a built environment that are occupied by users, and this research discusses the possibility of the impact of the place on its occupant’s health, whether that impact results from the site itself or from the shapes and proportions of the spaces in which people reside. It also demonstrates the methods of discovering places that have a positive or negative impact on people, in addition to the ways to enhance the body's Bioenergy, especially during the pandemic period.

Dr. Sam Osmanagich pointed to the ancients' knowledge of the great importance of the impact of the site's energy on humans and living beings, as they maximized the benefit from it by designing the shape of the building and the materials used in construction. He also added that “in the twenty-first century we now return to this forgotten knowledge. We have a chance to change our future for the better” [5].

## Bioenergy in the human body

The human body contains more than one kind of energy, which are essential for its life. One kind is the chemical energy produced by the body that occurs during the metabolism and the conversion of food into calories. Likewise, each person has a thermal energy that distinguishes him from the others, as each person has his own thermal signature. Matthew Stevens refers that a human male gives off about 100–120 Watts of energy, and 80% of a typical human's body power is given off as heat [6]. In addition, our bodies contain a small amount of natural radioactivity, as it contains radioactive isotopes of potassium-40, which emits beta particles and gamma rays [7]. Humans inhale natural radioactive elements that are present in the earth crust or produced by cosmic radiation. “These elements then start irradiating our bodies from the inside”, we are radioactive creatures [9].

It is known that our bodies contain an electrical energy; The muscles work according to its response to the electrical pulses produced by the brain passing through the nerves. Even dreams are related to an electrical activity inside the brain. We are electrical creatures, and our bodies are electromagnetic generators [7]. This paper focuses on the way the human body’s energy affected by the natural Earth’s energy resulting from the site, and the energy resulting from the shapes of the spaces we occupy, as it will be demonstrated later.

**Fig. 1:**
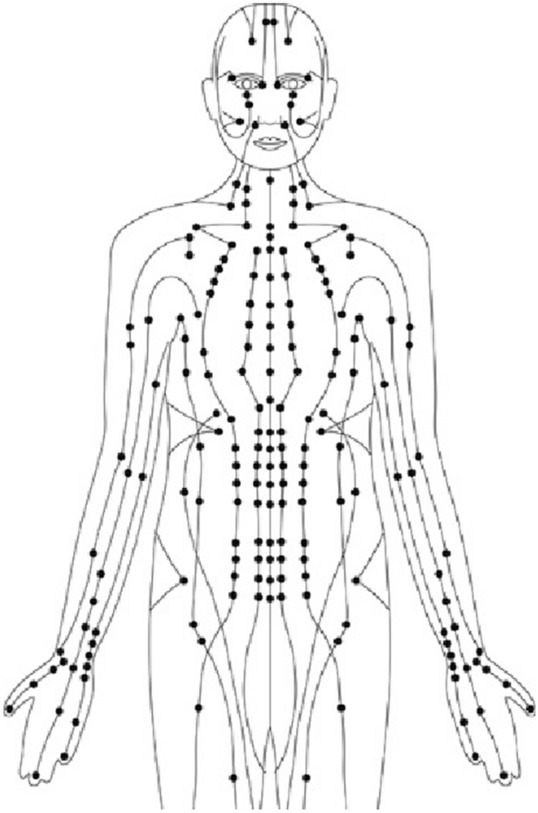
Meridians and acupuncture points on the human body. Author as adapted from Thurnell-Read [8]

The Ancient Chinese civilization presented a conception of energy in the human body, as it was described as paths (or Meridians) that pass through the human body. There are 14 Meridians that connect specific points on the skin, as shown in Fig. 1, and they reflect the health status of the internal organs. If the energy flow is blocked in one Meridian, it will negatively affect the wellness of the organs linked to it [10]. To remove that blockage, the acupuncture will be the treatment of this case.

**Fig. 2:**
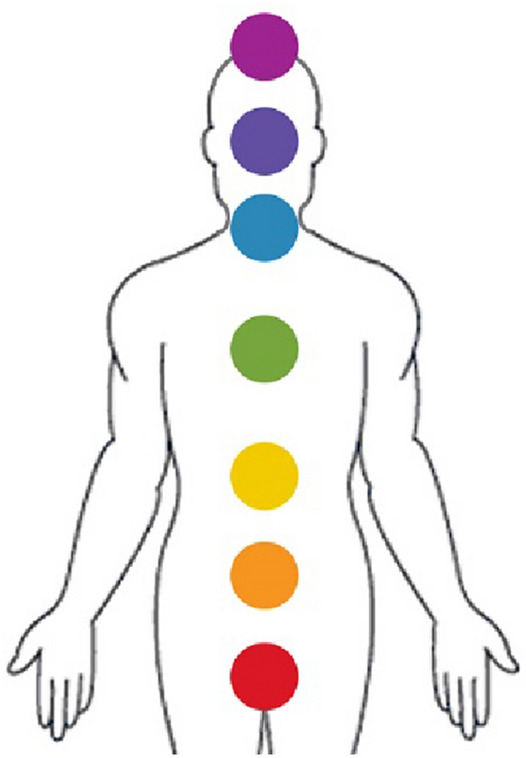
The 7 main Chakras in the human body. Author as adapted from Thurnell-Read [7]

According to Ancient Indian beliefs, the energy of the human body consists of seven main gates, which are called “Chakras”, through which energy passes to the body, and it also expresses the functions of the internal organs of the body. Some references mention the presence of secondary Chakras that work along with the seven main Chakras. Figure 2 shows the positions and colors of the main Chakras in the body.

Recently, energy in the human body has also been defined in various ways, such as the theories of "Subtle Bodies" and "Aura", which will be explained in the following part.

**Fig. 3:**
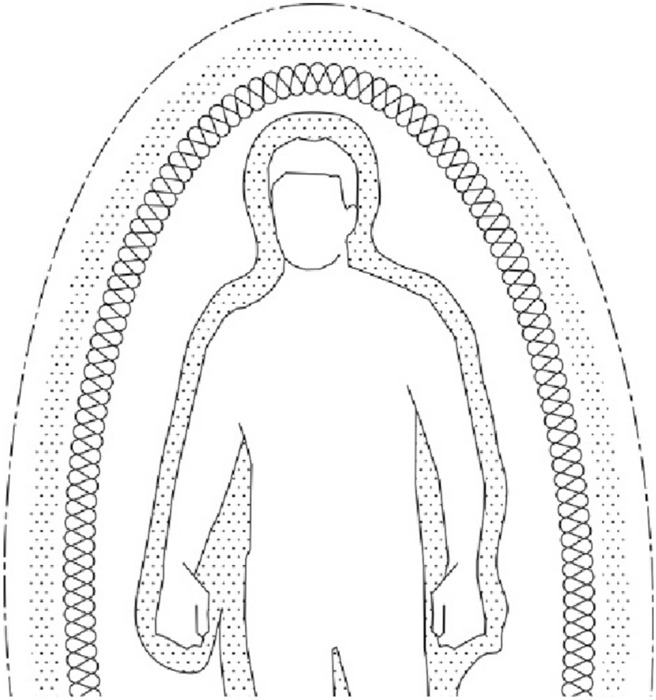
The subtle bodies theory. Author as adapted from Thurnell-Read [7]

Subtle Bodies Theory considers that the human body consists of five bodies of energy that contains each other, as shown in Fig. 3. The physical body is one of them, which is energy as well, because it is originally composed of molecules and atoms containing negative and positive charges, which are electrons and protons. Four other energy bodies envelop the physical body; they are like layers around each other. These bodies are called the etheric, emotional, mental, and spiritual bodies, and each has its own frequency. The physical body has the lowest frequency, so it is visible, unlike other invisible bodies. Thus, the spiritual body has the highest frequency [8].

The aura can be described as an energy consisting of electrons and photons, which containing the information of the object it originated from. It also could be described as an electromagnetic energy that emanates from objects. Everything has its own aura, whether it was human, animal, plant, or inanimate. The aura is not visible because its frequency is beyond the visible spectrum.

Mostafa Gadalla in his book “Egyptian Cosmology” mentioned that the ancient Egyptian medicine considers the humans as being in tune with the universe, and the body is a complexed vibratory system. It believed that “everything is in a constant dynamic state of movements which are intimately connected to the rhythms, harmonies and pulsations of the universe” [11].

It is clear from the above that the human body is not only a physical body, but a body with many different types of energies, and its electromagnetic energy has been described in various ways in different civilizations and eras as previously explained, and since this energy has an electromagnetic nature, then consequently the human body will be affected with the earth's electromagnetic energy, whether that effect is negative or positive. The following part demonstrates an explanation of the most important of those effects.

## The impact of the place on the human body

Humans knew previously and recently that they are affected both physically and psychologically by the place they occupy. This impact could be a result of many factors, and this research focuses specifically on two factors only, the earth energy and building shape energy, where the building shape is defined by its design and proportions. Alike the natural electromagnetic energy of the human body, emerging and surrounding it, the earth has almost the same concept. In addition, any building has a natural energy resulting from its shape, and can affect the humans’ body biofield, as it will demonstrated in detail in the following part.

## The impact of the location on the human body

By observing the different perceptions of electromagnetic energy in the human body, the author found that it is generally expressed either in the form of energy paths on the body or in the form of energy fields that interfere and surround the body. Interestingly this perception is very similar to the natural electromagnetic energy of the Earth, as it will be explained later. But before demonstrating the earth electromagnetic energy forms and its impact on the human body, we must first know the reasons of its emergence.

**Fig. 4:**
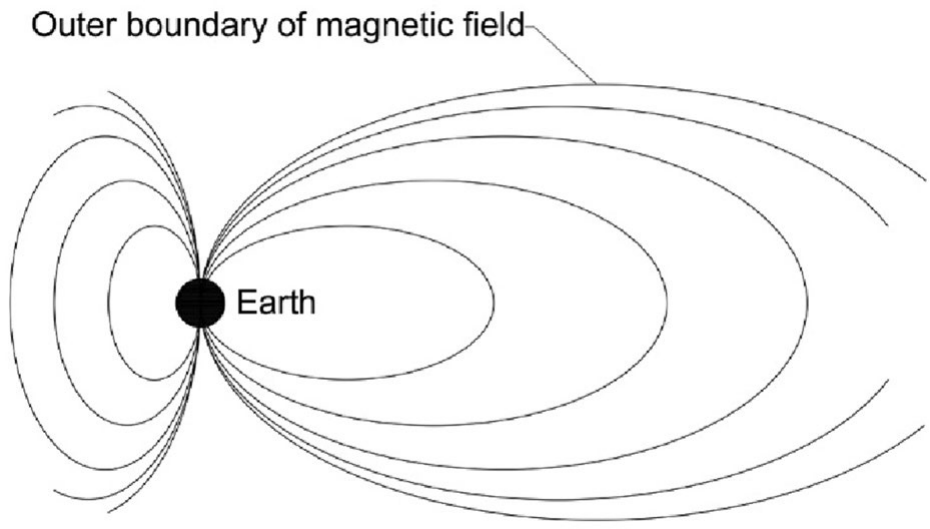
Earth's magnetic field. Author as adapted from Hall [16]

Most of the Earth's core consists of molten iron. The increasing pressure towards the Earth's center creates an inner solid iron core with 1200 km in radius. This metal core is the origin of the earth magnetic field [12], as the electric currents and the magnetic field of the earth are created by the flow of liquid iron in its core [13]. The process of regulating the electric currents in rolls around the north–south polar axis is the result of both the convection of molten iron within the outer liquid core and the Coriolis effect caused by the Earth's rotation around itself. Another supporting magnetic field is created by the flow of molten iron across the original magnetic field, making the Earth's magnetic field sustainable, which is called Dynamo Theory. Although the average magnetic field strength around the Earth's liquid core is 25 gausses, its intensity on the Earth's surface ranges between 0.3 gauss around the equator and increases to 0.6 gauss near to the poles. The Earth's magnetic field extends a few tens of thousands of kilometres into space [14], as shown in Fig. 4.

The Earth’s electromagnetic energy appears as lines, grids, spots, and other forms. These kinds and forms could be intersected in certain locations, where the word location in this research is generally refers to a position on the earth or site. This increases its impact, or sometimes eliminate each other. Therefore, the energy quality varies from location to another. Some have positive impact for humans and living beings, while some are negative. The earthly electromagnetic disturbances in some locations that has a negative impact on the human’s health is called Geopathic Stress [8]. The change of both earth magnetic field and radiation, geological faults, conductivity discontinuity caused by both subterranean material and water, are parameters that constitute a geophysical anomaly [15].

**Fig. 5:**
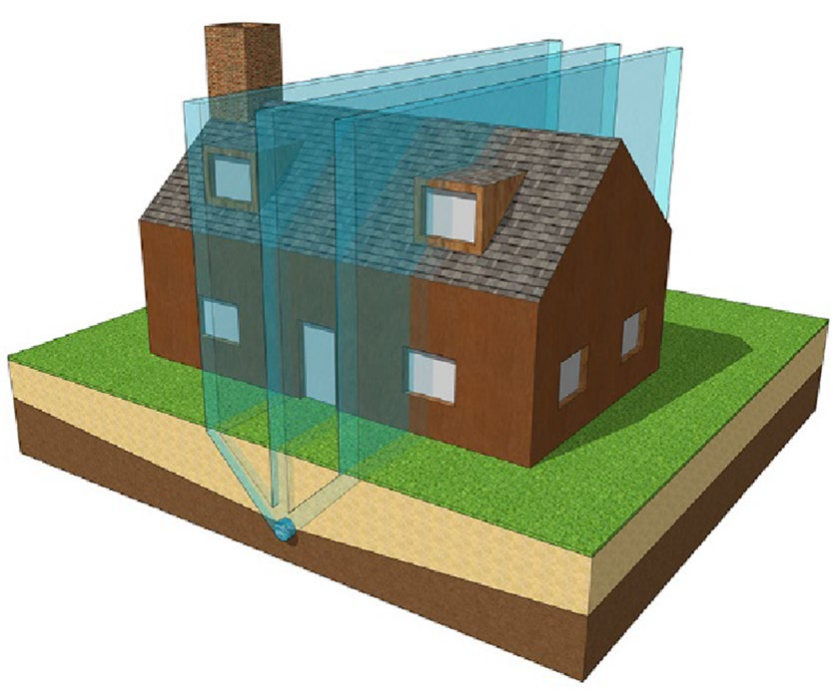
Radiation caused by the underground water streams. Author as adapted from Cowan and Girdlestone [17]

One kind of Earth’s natural electromagnetic energy is the radiation generated by underground water streams, and it is known that water is a good conductor of electricity. These radiations rising from beneath in the form of vertical vortices resulting from the flown underground water [16]. It appears in three parallel lines of vertical radiation [17], as shown in Fig. 5. Some of these radiations may be harmful to the humans and living beings above their streams, while others may be useful. Its impact can appear at high altitudes in the upper floors of the buildings, and it can be straight or curved according to their streams [8].

**Fig. 6:**
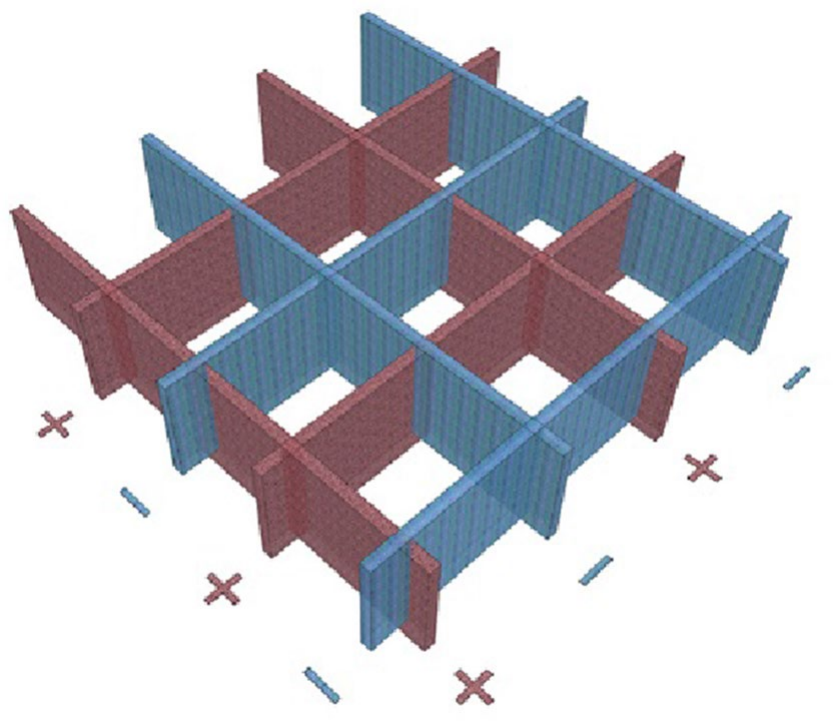
Imaginary representation of hartmann grid (Author)

The second most important kind of Earth’s natural electromagnetic energy are grids. There are many earthly energy grids such as the Hartmann Grid, which was first described by German physician Dr. Ernst Hartmann in the 1960s, where it appears in the form of invisible, radioactive vertical walls, and its lines run in an east/west direction with distances between approximately every 2 m, while the distances between its lines in a north/south direction range between 1.20 and 2.50 m. The lines of this grid carry negative and positive charges sequentially in both directions, as shown in Fig. 6. Another grid called Curry Grid, whose lines run at an angle of 45° in both directions [16]. In addition, there are two positively charged grids called Schneider Grid and the Second Schneider Grid [18]. The Author also discovered an energy grid along with Dr. Mohamed Samir El-Sawy in 2009 AD, which we named Alex Grid. The orientation of this grid is 23° 30′ west to the north direction and the distances between its lines are 25.5 × 40 m [19].

**Fig. 7:**
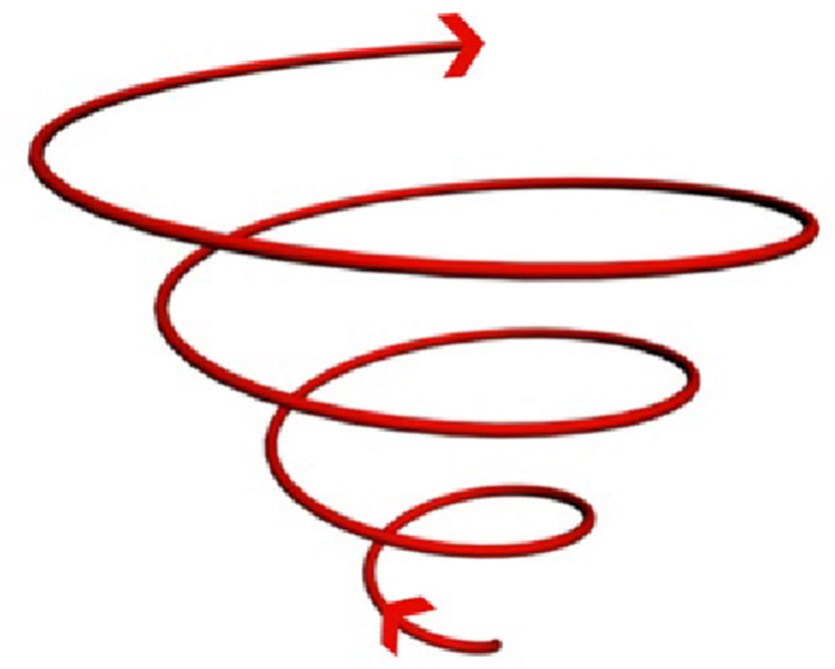
Positive energy vortex rotating clock wisely. Author as adapted from Karim [20]

The third kind of Earth’s natural electromagnetic energy is the Power Spots and Energy Spirals. Power spots are created when a group of positive energy paths, which are called Ley Lines, meet. When a power spot location is accompanied by an intersection of underground water streams, it creates a strong energy vortex in the form of an upward spiral rotates in a clockwise direction, as shown in Fig. 7. This kind of energy is often found at water wells which associated with healing properties [20].

There are many studies that confirm the negative impact on health when exposure to harmful underground water streams, as the negative impact increases for people who spend long periods in the location under which that harmful underground water streams. Cancer is one of the most famous of these harmful impacts on health, and the first documented study showing the relationship between harmful underground water streams and cancer was carried out by the German dowser, Baron Von Pohl, in his study of a small village in Vilsbiburg, Germany in 1929, after the spread of cancer cases in it. As he discovered through his research a 100% correlation between the sleeping places of cancer victims and these black streams that pass through the village [21].

For the Earth’s electromagnetic grids, some of them have a positive and others have negative impact on human health. For example, lines of Hartmann grid towards the north/south cause dampness, cramps, colic, and rheumatism, while the lines towards the east/west are associated with inflammations. Furthermore, sleeping above intersections of their negatively charged lines causes nervous disorders and headaches, and it gets worse if there were underground water streams in that location. However, the impact of some grids is positive, such as the positively charged Schneider Grid lines, which increase the thinking and communication abilities of man, and often found on pulpits in old churches or in lecterns in dining rooms of old monasteries. In addition, the second Schneider grid’s positively charged lines are associated with strength and physical healing. Its crossings are found on holy wells, chapels, shrines, castles, Roman forts, and ancient (prehistoric) standing stones [18].

Recently, studies that have been conducted on the geopathic stress zones confirm that they have a negative impact on human physical and mental health. As its effect can be explained through the presence of magnetite, iron, and water in human body, as well as the brain, heart, neural network electromagnetic fields [15].

For example, there is strong evidence that buildings located in geopathic stress zones can cause miscarriages and fetal abnormalities cases [22]. One of the experiments that were conducted on a group of people in such locations indicates weakness and disturbance in the body's energy, which is often related to the lymphatic system, the cardiovascular system, and the pineal gland [23]. A second study indicates that being in such places increases blood pressure, heart rate, and reaction time of humans [24]. Another study also indicates the possibility that the immune system is negatively affected when exposed to geopathic stress, and it causes a feeling of exhaustion, depression, and nervousness [25], which constitutes a greater threat in case of exposure to a viral pandemic, and it worsens the humans’ psychological bad state, as they are forced to stay at home due to curfew. Finally, a study indicates that staying or sleeping in those locations for long periods causes many symptoms that vary from fatigue, sleep disturbances, migraine headaches, rheumatism, asthma, and ending with Cardiovascular circulation problems and even cancer [26].

## The impact of the building shape on the human body

Dr. Sam Osmanagich pointed to the ancients' knowledge of the importance of strong energy sites on this planet, and that they used the tremendous force resulting from the pyramid, sphere, and sacred geometry shapes, in addition to the presence of iron that generates electromagnetism and quartz that enhances these energy fields, altogether provided them with clean and free energy. He also indicated that the studies on the pyramids that are being built since the nineties of the twentieth century until now in Russia, are based on studying the impact of its energy on human health and improving his spiritual senses, as well as on the electrical conductivity of materials, improving oil production, changing the weather, and reducing devastating earthquakes, volcanic eruptions, and the Ozone depletion [5].

The word “Geometry” is based on two Greek words, the word “Geo” means the earth and the word “Metry” means the measurement. Therefore, it literally means “earthly measurements” [27]. The concept of sacred geometry mentioned in the previous paragraph expresses the ancient belief that the mathematical relationships of shapes and designs lie behind the formation of all creatures, as they are not inspired by the human mind, but rather an extrapolation of the underlying structure patterns within beings in nature. The most famous representation of this concept is the Great Pyramid of Giza, which contains many proportions of sacred geometry.

Scientists Bill Schul and Ed Pettit tell us about their experiment about the effect of the pyramid shape, where two vessels of equal size and shape were filled with milk with one placed inside the pyramid and the other outside it, and after six days it was found that the milk inside the pyramid had turned into a group of separated layers that contain sour and curdled milk and a watery liquid, while some rottenness appeared on the surface of the milk in the other vessel, it was disposed of, then they took out the vessel from the pyramid and put it in the same place of the disposed vessel, and after six weeks the milk turned into a firm fatty substance with a taste of yogurt without any rottenness [28].

Dr. Volodymyr Krasnoholovets and Dr. Mykola Yatsuta conducted many experiments on their pyramid, which is made from metal frame and slate plates. For example, they put cabbage seeds for 1–3 days inside the pyramid, they found that its crop increases up to 50%, and when they covered the pyramid with metal plates, the fields inside the pyramid increased around 20–30%. They found that if a person sits inside their pyramid for 10–20 min, he will begin to feel dizzy, sensitive people feel a kind of powerful pressure on the head as they go inside. Dr. Krasnoholovets believes that the pyramid affects the fluidity of lymph and blood, and he suggests that all fluids in the body acquire a kind of a superfluidity [28].

The French scientist Antoine Bovis also discovered that the animals that crept into the king's burial room in the Great Pyramid and died in there, their bodies did not rot, but rather had kind of mummification. So, he built a small pyramid with the same proportions of the Great Pyramid and placed it towards the north/south magnetic direction as well. Bovis placed a dead cat in the same position as the king's burial chamber in the Great Pyramid, he discovered that the cat’s body did not rot, but was mummified [29]. This indicates that the pyramid has drying properties.

Dr. Volodymyr Krasnoholovets conducted numerous experiments with his colleagues from the Institute of Physics in Kiev, Ukraine on 17 large pyramidal shapes with greater acute angle than the Great Pyramid of Giza. These pyramids were made of fiberglass with different sizes and distributed in eight locations in Russia and Ukraine, where scientists in both countries have conducted many experiments using these pyramids in the fields of medicine, environment, agriculture, chemistry, and physics [30].

Professor Klimenko and Dr. Nosik from the Ivanovsky Institute of Virology Russian Academy of Medical Sciences studied the effect of the pyramid shape on "Venoglobulins", which are antibodies that protect us from virus infections that may enter our bodies. They placed the Venoglobulins inside the pyramid for several days, then they took a specific type of virus from an experimental mouse. They found these venoglobulins inhibited viruses breeding by three times more than the control group. That gives the possibility to strengthen the body's immune system against viruses [31], which constitutes a great opportunity in case of exposure to a viral pandemic.

A psychological study was conducted on 5000 prisoners from a prison in Russia, where some inmates were given solutions that were placed inside a pyramid, and within a short period of time, the violent behavior of this group disappeared compared to the control group. Other studies conducted on alcoholics and drug addicts showed that when they were given doses of glucose by injection or distilled water orally, which were previously placed inside a pyramid, a significant improvement occurred in their fight against addiction. These experiments and studies show the effect that the pyramidal shape can influence mental processes [32].

A group of researchers working at BMS College of Engineering in Bangalore, India, conducted a study on a group of people to find out the effect of the “Maitreya-Buddha” pyramid on their brain activity while practicing Meditation inside it. The EEG patterns were studied for 15 people, where a set of measurements outside the pyramid was taken before meditating and compared with same set of measurements after meditating inside the pyramid. The analysis of the results showed an increase in the Mean value of alpha brain waves for 13 people, while it decreased for only 2 people, and the amplitude of “Theta” brain waves showed an increase for 14 people, while it decreased for only one person, therefore the increase in both Alpha and Theta brain waves reflecting a state of relaxation after practicing meditation inside the pyramid compared to the meditation outside it, which confirms the effect of the pyramid on the brain’s state [33].

The science of “BioGeometry”, which began to appear in the nineties of the twentieth century, as this science was established in Egypt by Dr. Ibrahim Karim, after more than thirty years of research. This science is concerned with studying the effect of geometric shapes on the biological processes within living beings, the term “BioGeometry” consists of two parts “Bio” which is related to biological processes and “Geometry” which is related to shapes and formation [34]. The formation in BioGeometry is based on applying what is known as the balanced energy to the design through a group of formation and design basics, as these basics generate a special energy quality with a biological impact capability, which spreads within the designed spaces [35]. The following parts indicate what confirms the physical impact of shapes on the human body.

In April 1999, the "BioGeometry Consulting Ltd." has been invited to participate in the "The National Liver Disease Research Project" at the Pharmaceutical faculty of El Azhar University, where a comparative research was conducted between all treatments through traditional and alternative medicine for “Hepatitis C” virus. Where the results of the first phase, which were conducted on about 300 patients, demonstrated an improvement in more than 90% of cases, after placing an BioGeometrical coated aluminum pendants within the vital field of patients’ body energy. These pendants contained engraves of specific designed geometric shapes”BioSignatures”, while the rest of the treatments achieved results ranging between 20 and 50% [36]. It is a great opportunity to conduct a research to assess the efficiency of BioGeometry against other viruses.

A research project was established under the patronage of the Swiss Mediation Authority for Mobile Communication and Environment, with collaboration of governmental telecom provider “Swisscom” to treat health problems resulting from electro-sensitivity in the village of “Hemberg” in Switzerland in 2004. A BioGeometry energy balancing shapes was implemented to remedy ailments of electro-sensitivity in this village, which has achieved success in removing diseases caused by electro-sensitivity and healing many other health conditions, in addition to the positive impact on the general environment of the area, later the project was named “The Miracle of Hemberg” after reports Issued by official bodies and independent studies [37]. In 2005, the local government of the town of "Hirschberg" commissioned Dr. Ibrahim Karim to implement similar solution that succeeded in the village of Hemberg. The BioGeometry shapes succeeded again to alleviate the electro-sensitivity symptoms of the village residents. It also succeeded in improving the health and productivity of livestock in the village, which is an important economic source for the population, as this experiment was referred to in the documents by "Electro-smog: The Miracle of Hirschberg" [20].

## Methods of discovering the positive or negative energy in the place

There are many traditional ways through which earth energies can be discovered, which depends on the human body, as this can be done through kinesiology, dowsing, sensing or through observing the behavior of living beings in the place [38], where some living beings prefer to be in places of positive energy for humans, such as horses and dogs, and others like cats, beetles and ants prefer to be in places of negative energies for humans, as these places are more suitable for the bodies and energies of those creatures [8].

**Fig. 8:**
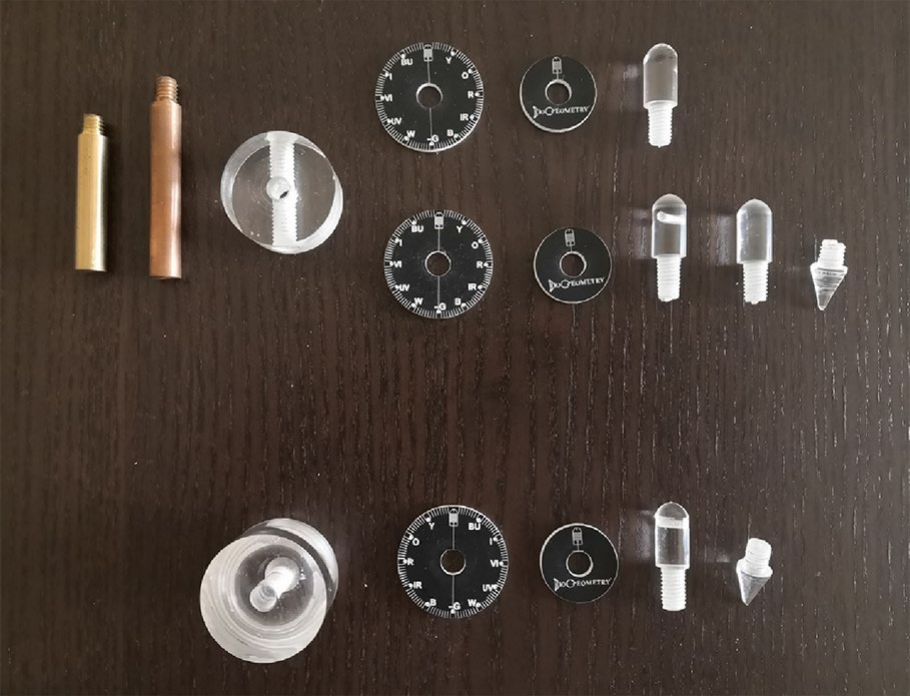
Two of BioGeometry pendulums, the vertical dial pendulum components (up), and the horizontal dial pendulum components (down), (Author)

Through kinesiology, it is possible to know the impact of the place, negatively or positively, by testing the body muscles, which is linked to the electrical signals emitted by the brain, and thus the effect of the place on the human body energy field can be assessed. There is also another traditional method that has been used in ancient and modern times to detect the locations of earth energies, which is called “Dowsing”, while the French priest “Alex Bouly” named it “Radiesthesia” [39], which is a word of Latin origin meaning sensitivity to radiation, as it is a science of using man’s sensitivity to vibrations to obtain information from living beings or objects by creating resonance with their energy fields, using specially calibrated instruments [40]. There are two types of radiesthesia, one of which is mental radiesthesia, and the other is based on the principles of physics, and both work by transforming and amplifying the vibrations flowing through the human’s body, which are translated by the movement of the detector which can be pendulum or rod [41]. BioGeometry has developed pendulums that are specifically designed to measure both horizontal magnetic waves and vertical electric waves of earth energy grids, such as Hartmann and Curry grids. These pendulums which shown in Fig. 8, are based on the principles of physics radiesthesia, and are used by the author as a practitioner and researcher.

The modern devices that can be used to detect the earth energy vary according to the way they work. Some of them depend on measuring the condition of the human body to determine whether it is affected positively or negatively by the location in which it is in. Other measures the energy of the location directly, through digital measuring devices that can measure tiny variations in the earth’s magnetism and electricity from one place to another. However, modern devices often rely on traditional methods for detecting energy locations primarily before quantifying them.

The "Bio-Well" is one of the devices that depends on measuring the energy of the human body, which depends on the analysis of electro-photonic emissions resulting from exposing the fingertips to a weak electrical current for less than a millisecond. As a response to this stimulus, an electron cloud is made up of light energy photons, through which it is possible to analyze the condition of the body in general, as well as each of the internal organs in detail [42]. It is also possible to use the "ETAScan" device, which depends on analyzing the human's bioenergy, as it captures the human body as an energetic structure and recognize its internal organs as a “frequency swarm” that has a specific structure. As every organ has its own vibration pattern, changes in these vibrations allow precise determine of the organ’s functionality [43]. The device sending low emission of laser to the body and receives the internal organs response through a specialized neck sensor, then it analyzes the results [44].

For location measuring devices, there are devices that can measure energy vibrations; electric or magnetic energy charges, radio waves, as well as radioactivity in a specific location. For example, a 3D geo-magnetometer was used in one research for measuring terrestrial magnetic field within a residential building to investigate the Influence of Geomagnetism on the building occupants [45]. In another research an Esmog Spion and a magnetometer was used to measure the earth electrical and magnetic fields respectively [46]. A “Geo-resistivity meter” can be used to detect anomalies in the Earth energy, but it is suitable for open spaces with large areas [24].

It is also possible to use devices that uses the “Light Interference Technique” method, which depends on using a laser light source and placing a receiver at a distance from it. In case geopathic stress is present in this area, it will interfere with the laser, then changes in the current will be determined [47].

Using the same idea but in another way, the “Full-Point” company has developed a device can detect Earth energy grids, such as the Hartmann and Curry grids. Their method depends on detecting changes that occur in the “FM” waves, using two devices, one of them sends the waves are called “Sounder”, and the other receives them are called “Sonar” [48]. This device can be used inside small spaces in buildings.

## Ways to enhance the body's bioenergy

One of the eight Legal Maxims Related to the Higher Objectives of Islam is “preventing harms is to be put forward before brining benefits” [49]. Therefore, the priority is to stay away from natural energy sources that cause health problems, especially if these sources are found in people’s usual sleeping or sitting places. The longer a person is exposed to negative energy sources, the more affected he will be. When a person notices that a certain place is not desirable for him without a clear reason, or if he felt fatigue or headache after sitting in that place for a long time, it is better to move away from that place and change it. When a person suffering insomnia or feels tired when wakes up, the same action should be done to his sleeping place, where that indicates the negatively energy quality that affected the person. Therefore, it is recommended to rearrange the furniture in the house as much as possible to avoid harm that may affect the body’s bioenergy, as well as its physical and psychological health.

Mapping of negative global grid lines in the residential districts allows avoid their negative impact and improve the health of the community members [18], while the researcher suggests that it is better to map positive energy grids as well, in order to gain benefit side by side avoiding their harm, and thus improve the health of community members furtherly.

David Cowan demonstrates in his book “Safe as Houses?” several solutions that can be applied to deal with different types of negative earth energies. Although it is preferable to avoid places of negative energies when choosing places to sit or sleep, in the case of existing of negative earth energy grids intersections, quartz crystals can be used, which change the negative quality to become positive, but it is required to clean the crystals continuously. In the case of negative underground water streams. It is recommended to plant “L” or “V” shaped iron or copper rods in the outer courtyard of the house. In the case of a negative energy vortex, a copper coil of at least 10 turns can be placed in its center, a large coil size is preferred to change the negative energy quality to positive. Mirrors also can be used to change the directions of undesirable energy paths [17].

Through the experience of the physicist Alan Hall in dealing with natural and artificial electromagnetic fields, he has developed a device that can produce a protecting field against the negative influence of electromagnetic fields, whatever their source is. On putting this device in a building produces what is called a “Biodynamic field”, which protects humans and living beings from the harmful effect of the electromagnetic fields [16].

Moustafa Gadalla believes that human healing includes both his body and his soul, based on the writings of Herodotus in the fifth century BC about the Egyptians, as the happiest, healthiest, and most religious race. Ancient Egyptian medicine considered man as a whole; in tune with the universe, as the human body is an extremely complex vibratory system, which is intimately connected to the rhythms, harmony, and pulsation of the universe. If the rhythmic patterns of a person are disturbed, this will be an indication of health problems in the future, and to heal a person, he must be brought back into harmony with this universe. Colors, numbers, and metals that correspond to these universal rhythms were used to adapt these harmonic forces, in addition to using certain words with proper tones in several treatments [11].

Dr. Mohamed Elsawy in his book “Energy and the shape language”, suggests a more comprehensive methodology approach to improve the energy of the place, and the ways to deal with the existing energy problems to improve the bioenergy of the human body. This methodology is based on three axes, each of which includes three levels [34]. The author briefly demonstrates that methodology as shown in Table 1.

**Table 1:** A brief of Dr. Mohamed Elsawy methodology for improving the energy of the place and ways to deal with the existing energy problems, Author

The first axis: Site study			
Level 1	Site and surrounding environment	Notes	Topography analysis—Observation of beings
Level 2	Earth energy		Positive and negative energy grids and spots
Level 3	Soil components		Existing metals and radiation rates
The second axis: Design processes			
Level 1	Design grids	Notes	Its relation to the Earth energy grids
Level 2	Building location and orientation		Its position and relationship to cardinal directions
Level 3	Forming using BioGeometry		Applying the balanced energy
The third axis: Executive designs			
Level 1	Building material specifications	Notes	Its relation to the body’s health and energy—Natural materials are preferable
Level 2	Utility networks		Study the electricity, water, and sewage paths
Level 3	Interior finishes		Choosing colors and furniture placing

Ibrahim Hegazy and Sherif Sheta made a study on a 3D six digital models with different forms and materials to investigate their influence on the users’ brainwaves. With the mean of CST Microwave Studio, they identified the resonance frequency of these forms, they concluded that each model generates its own resonance frequency, according to its form and material. These resonances cause changes in the human brainwaves and consciousness [50].

The BioGeometry design methodology relies on nine main pillars, which provide us with tools that can enhance the design criteria to improve the energy of the place, by infusing the natural harmony in the subtle energy qualities, and thus the energy of the person who occupy. The author briefly demonstrates that methodology as shown in Table 2.

**Table 2:** A brief of BioGeometry design methodology to improve the energy of the place, Author

	BioGeometry design pillars	Notes
1	Earth Energy Design	Design in harmony with various forms of the Earth’s energy
2	Sky-linked Design	Connect the building with the positive energy spots in the sky
3	Qualitative Harmonics	Generating positive energy using numbers, ratios, angles, etc.
4	Design principles	Using BioGeometry in both building and furniture design
5	Motion in Design	Using movement to generate positive energy—The BioGeometry Energy Key
6	Qualitative Global Scaling System	Energy points and rings surrounding the design element
7	Archetypal design codes	Subtle energy patterns that support the physical shape energy functions of all living beings
8	Material Energy Quality Balancing	Changing the subtle energy quality of any material to infuse the place with the harmonizing energy quality
9	BioSignatures	Using BioSignatures to enhance the energy of the human body

Dr. Ibrahim Karim Designed a Special Needs Integrated community Centre, which is specially designed for people with nervous disorders, depression, MS, ADHD, ADD, autism and other brain problems, according to the BioGeometry methodology. This project relied on the fact that the design itself plays a major role of reducing the stress in the brain, where the regular orthogonal and circular shapes in our built environment can increase the problem. Previously a Ph.D. thesis in Cairo University included a pilot study by build a single class to test and ensures the BioGeometry methodology. Fortunately, the measurements showed successful results, as it showed a positive effect of BioGeometry solutions on children with special needs.

## Conclusion

The human body contains more than one type of energy necessary for his life and living, such as chemical energy, thermal energy, and electric energy, as well as a small amount of radioactivity. The energy of the body was expressed in Ancient China as paths on the skin, and in Ancient India as seven gates distributed in the body, and recently as subtle bodies surrounding and penetrating one another or that each body surrounded by its own energy field. The body seems to be a very complex vibrational system closely connected with the rhythms, harmonies, and pulsations of the universe.

The Earth's natural electromagnetic energy may exist in the form of lines, grids, spots, or others, and it affects positively or negatively humans and living beings within its area. The building has natural energy resulting from its shape, which can affect the energy field of the human body, as the ancients knew this fact, and it has been proven recently through the scientific experiments.

There are many traditional methods through which the location and influence of earth energy can be discovered, starting from observing the behavior of living beings, sensing, or using kinesiology. There are modern measuring devices that depend either on measuring the energy of the human body or measuring the energy of the site.

There are many methods and theories to deal with the energies of the place that have a negative impact on humans, starting from the avoiding it, or using some materials and tools to change its pathway or reverse its impact to become positive, as well as using of devices that produce a positive energy quality that eliminate the negative impact of the place. Finally, the use of comprehensive methodologies to deal with the negative energies of the place by applying a regulatory energy, that eliminates the negative impact of the existing energies, whether were natural or artificial.

## Data Availability

The datasets generated during and/or analysed during the current study are available from the corresponding author on reasonable request.
